# Evaluation of lung function and airway impedance in response to ultra-marathon

**DOI:** 10.3389/fphys.2026.1840102

**Published:** 2026-08-03

**Authors:** Kelsey E. Joyce, Courtney M. Wheatley-Guy, Jesse C. Schwartz, Glenn M. Stewart, Nicholas B. Tiller, Caitlin C. Fermoyle, Bryan J. Taylor, Robert Murdock, Keren Constantini, Paul Robach, Alice Gavet, Loic Chabridon, Pierre Bouzat, Bruce D. Johnson

**Affiliations:** 1 School of Sport Exercise and Rehabilitation Sciences, University of Birmingham, Birmingham, United Kingdom; 2 Division of Cardiovascular Disease, Mayo Clinic- Arizona, Scottsdale, AZ, United States; 3 Birmingham Medical Research Expeditionary Society, Medica School, University of Birmingham, Birmingham, United Kingdom; 4 Divison of Cardiovascular Disease, Mayo Clinic- Rochester, Rochester, MN, United States; 5 Sydney Medical School and Charles Perkins Centre, Faculty of Medicine and Health, University of Sydney, Camperdown, NSW, Australia; 6 Lundquist Institute at Habor- University of California, Los Angeles, Institute of Respiratory Medicine, and Exercise Physiology, Torrance, CA, United States; 7 Department of Respiratory Medicine, Royal Prince Alfred Hospital and University of Sydney, Sydney, NSW, Australia; 8 Division of Cardiovascular Disease, Mayo Clinic- Jacksonville, Jacksonville, FL, United States; 9 Cardiology – Community Medical Center- Missoula, Bozeman Deaconess Health Services, Bozeman, MO, United States; 10 Sylvan Adams Sport Science Institute, Tel Aviv University, Tel Aviv, Israel; 11 Ecole Nationale des Sports de Montagne, site de l’Ecole Nationale de Ski et d’alpinisme, Chamonix, France

**Keywords:** forced oscillation technique, high altitude, lung function, trail running, ultra-marathon

## Abstract

**Introduction:**

Ultra-marathon races present a prolonged cardiopulmonary physiological stress but the magnitude, persistence, and impact of race environment on respiratory function are incompletely understood. The aim of this study is to observe the effect of ultra-marathon trail races on both lung function and airway impedance.

**Methods:**

Fifty-seven individuals (49/8, M/F) participated in either the Courmayeur-Champex-Chamonix (CCC^®^, 101.5km), Ultra Trail du Mont-Blanc (UMTB^®^, 171.5 km), or the Hong Kong 100 (HK, 100 km) races. Participant demographics were measured pre-race and included: age (40.7±10.3 yrs); height (175.5±7.1 cm), body mass (69.7±8.3 kg). Spirometry, airway impedance (via forced oscillation technique, FOT), maximal inspiratory (MIP) and expiratory pressure (MEP), and exhaled nitric oxide (ExNO) were assessed before, immediately (18–4 hours after), and 188–24 hours post-race. Mixed effects analysis with Dunnett’s post-hoc correction were conducted to evaluate the effects of race and time.

**Results:**

Results demonstrated significant effects of time for: forced vital capacity (FVC), forced expiratory flow over one second (FEV_1_), peak expiratory flow (PEF), forced expiratory flow between 25-75% (FEF25-75), forced expiratory flow at 25% and at 50% (FEF_25_ and FEF_50_, respectively), MIP, MEP, airway reactance (X) at all frequencies from FOT (X_5_, X_11_, X_19_), respiratory rate, and ExNO (all *p*<0.05). A significant effect of race was also observed for ExNO (*p*=0.002).

**Discussion:**

Findings indicate declines in lung function and airway impedance immediately after ultra-marathon races, some of which, persisted up to 24 hours post-race. Further investigations are required to better understand the etiology of changes in lung function following ultra-endurance events.

## 1 Introduction

Ultra-marathon races, which involve running distances greater than 42 km (26.2 miles), have rapidly increased in popularity and difficulty in recent decades (Scheer, 2019). These racing distances created a prolonged period of physiological burden. In addition to the effects of prolonged strenuous exercise, many of these races often include extreme environmental conditions such as heat, cold, high altitude, and variable air quality. Sustained hyperventilation, airway drying, hypoxic exposure, and inhalation of airborne pollutants may collectively contribute to airway inflammation, altered airway mechanics, and respiratory muscle fatigue. Furthermore, environmental stressors such as altitude may increase the risk of pulmonary complications via exacerbation of hypoxic pulmonary vasoconstriction, which can lead to high-altitude pulmonary edema and acute mountain sickness. It is clear that ultra-endurance races result in a substantial respiratory stress (Wüthrich et al., 2015), but the magnitude, persistence, and impact of race environment on respiratory function remain unclear. Ultra-marathon events therefore represent a natural model of graded environmental and ventilatory stress, allowing for evaluation of how cumulative exposure influences pulmonary physiology.

Previous work evaluating the effects of ultra-endurance race participation have shown mixed results, with some studies finding a decline in spirometry and respiratory muscle fatigue, while others have concluded minimal to no impairment (Mahler and Loke, 1981; Warren et al., 1989; Rogers et al., 2002; Wüthrich et al., 2015). Such variability may be driven by differences in race characteristics such as distance, altitude exposure, temperature, and air quality. Additionally, the use of primarily spirometric measures may lack the sensitivity to detect subtle or more peripheral airway dysfunction. Collectively, this highlights that to overcome these limitations, there is the need to understand the influence and interplay of race-specific environmental stressors and use more sensitive physiological assessment to fully characterize the respiratory changes resulting from ultra-endurance competition.

The forced oscillation technique (FOT) in contrast to spirometry is an effort-independent assessment of not just airway obstruction or resistance (R), but also airway mechanical properties (X), and the use of different frequencies can evaluate localized changes in the central, intermediate, and peripheral airways (Zerah et al., 1995; Oostveen et al., 2003; Brashier and Salvi, 2015). Because of these differences from spirometry, FOT presents a sensitive way to measure subclinical changes in airway tone, compliance, and heterogeneity that may be exhibited following prolonged endurance activity (Oostveen et al., 2003; Gibson-Alves et al., 2022). Similarly, the measurement of exhaled nitric oxide (ExNO) has been shown to be an indirect marker of airway inflammation associated with nitric oxide synthase (NOS2) activity (Dweik et al., 2011). We have also seen declines in ExNO at altitude as a marker of acclimatization, which we have hypothesized is related to eNOS metabolism to facilitate pulmonary vascular dilation to counteract hypoxic pulmonary vasoconstriction in the lungs (Annesi-Maesano and Dinh-Xuan, 2016; Summerfield et al., 2018). As such, changes in ExNO are likely influenced by prolonged exposure to cold, altitude, and pollution, and may help differentiate inflammatory from mechanical mechanisms underpinning transient changes in lung function with ultra-endurance participation. The objective of this study is to evaluate changes in lung function using traditional methods of spirometry and respiratory muscle strength, alongside FOT and ExNO following three ultra-marathon races that varied in distance (100 km vs. 100 miles), altitude exposure, temperature, and humidity conditions. By examining races that represent differing levels of cumulative ventilatory and environmental stress, we sought to characterize the transient physiological impact of ultra-endurance competition on airway mechanics and inflammation. We hypothesized that ultra-marathon race participation will result in a significant decline in lung function and impaired airway mechanics that persist up to 24 h, with the magnitude of exacerbation greater in races with higher altitude exposure and longer distance.

## 2 Methods

### 2.1 Ethical approval and consent

This study was part of a larger study that was approved by both the Mayo Clinic Institutional Review Board and the Comite de Protection des Personnes (CPP) Sud-Ouest Et Outre-Mer II, France. All participants gave written informed consent prior to participation, and every aspect of the study conformed to the Declaration of Helsinki 2013 (except database registration) and Health Insurance Portability and Accountability Act (HIPAA) guidelines.

### 2.2 Participants and race characteristics

**Figure 1:**
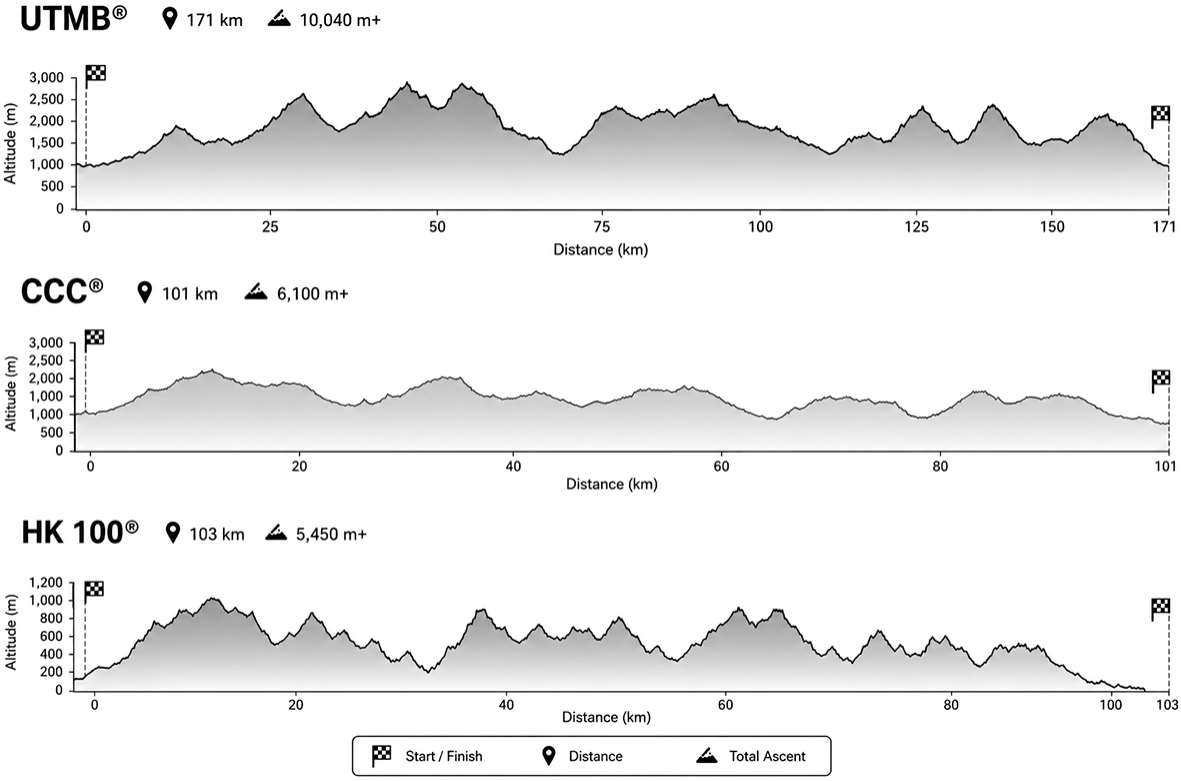
Ultramarathon race profiles. Ultra-Trail du Mont Blanc (UTMB) (top), Courmayeur-Champex-Chamonix (CCC) (middle), and Hong-Kong 100 (HK) (bottom). Race altitudes are denoted on the y-axis with race distances in km shown on the x-axis. The UTMB CCC, and HK races had starting elevations of approximately −1,035, 1,224, and 60 m; racers climbed to peak elevations of approximately 2,565, 2,565, and 957 m and covered a total elevation gain of approximately 10,040, 6,100, and 5,450 m, respectively.

A total of 73 participants were initially recruited; however, individuals were excluded from analysis if they did not complete more than 80% of the race (due to time limit or other causes) or did not come back for the immediate post-race visit (due to malaise, unavailability, or withdrawal). Fifty-seven adults were ultimately included and completed either the 101.5-km Courmayeur-Champex-Chamonix (CCC^®^) (*n* = 17) starting in Courmayeur, Italy; the 171.5-km Ultra Trail du Mont Blanc (UMTB^®^) (*n* = 22) starting in Chamonix, France; or the 100-km Hong Kong 100 (HK) (*n* = 18) race starting in Hong Kong, China. Race ascent profiles are presented in **Figure 1**. UTMB and CCC races included mountainous trail running with both having multiple high-altitude passages (>2,500 m) in extremes of temperature and humidity [−6 to 28 °C and 35% to 75% (2018) and 6 to 29 °C and 35% to 70% (2019)]. By contrast, the HK race elevation remained near sea level with less extreme temperatures (15.6 to 18.9 °C and 78%–94%).

Some data from this study (e.g., ExNO) have been published elsewhere with a different *a priori* question in mind (Tiller et al., 2022; Parks et al., 2023; Stewart et al., 2024; Chou et al., 2025). For those studies, the objective was to evaluate sex differences blood biomarkers and lung function in finishers and the effect of ultramarathon racing and differences on race altitude on alveolar–capillary recruitment and lung diffusion. For sex-specific analysis, data were limited to eight female finishers and compared to eight time-matched male finishers. For evaluation of lung, diffusion this analysis was performed on the 67 runners who returned to completed measurements at all time points (pre, immediately post, and 24 h post) regardless of whether they finished the race. The current manuscript is focused on all participants who finished at least 80% of their respective race and aims to evaluate the change in lung function and impedance following the ultra-endurance race, which has not been presented in previous publications that used different stratifications and had a different focus (Stewart et al., 2024; Tiller and Illidi, 2024).

### 2.3 Experimental design

Participants reported to a temporary laboratory [École Nationale de Ski et d’Alpinisme (ENSA) for the UTMB and CCC and a local hotel for the HK] for baseline measurements taken 24–72 h prior to the race start, immediately post-race (within 1–4 h), and 18–24 h after race completion.

### 2.4 Measurements

#### 2.4.1 Anthropometrics

Height (at baseline only), body mass, and body composition (% body fat and lean mass) were measured or calculated at each visit. Measurements of body fat were assessed using a Maltron Bioelectrical Impedance Analyzer (Rayleigh, UK) for CCC and UTMB races. Bioimpedance measurements were measured while individuals were laying supine on a bed.

For HK, body fat was assessed using the Phillips Lumify ultrasound with a 12-MHz linear array transducer (Phillips Healthcare, Andover, MA) together with the seven-site area calculation (triceps, subscapularis, chest, midaxillary, suprailiac, abdomen, and thigh) (Wagner et al., 2016). For the seven-site measurements, individuals stood in a neutral position, while the probe was placed in the proper sites with images captured and saved for later analysis using MuscleSound^®^ software (Glendale, CO).

#### 2.4.2 Spirometry and respiratory muscle strength measurements

Participants performed slow vital capacity (SVC) and forced vital capacity (FVC) maneuvers using a calibrated digital spirometer [Medical International Research (MIR), Spirodoc spirometer, New Berlin, WI and CPFD/D USB Spirometer, MGC Diagnostics, St. Paul, MN] according to standard procedures (Miller et al., 2005). Forced expiratory volume in 1 s (FEV_1_), peak expiratory flow (PEF), and forced expiratory flow at 25%–75% (FEF_25–75_) were analyzed from spirometry tests. Exercise-induced airway obstruction (as measured from spirometry) was also assessed and was classified as at least a 10% reduction in FEV_1_ from baseline (pre-race) (Vernillo et al., 2015).

Maximal inspiratory pressure (MIP) and maximal expiratory pressure (MEP) were measured using a handheld pressure meter (Micro RPM system, Vyaire™, Mettawa, IL) according to standard procedures (American Thoracic Society/European Respiratory, 2002). Each Mueller maneuver (for MIP) was initiated from residual volume, whereas each Valsalva maneuver (for MEP) was initiated from total lung capacity. Participants repeated the maneuvers within standard practices of American Thoracic Society/European Respiratory Society recommendations (American Thoracic Society/European Respiratory, S, 2002; Costa et al., 2010). Because of equipment availability, MIP and MEP were measured at the CCC and UTMB only.

#### 2.4.3 Forced oscillation technique measurements

Airway obstruction and mechanical properties were measured via FOT with a Resmon Pro^®^ (MCG Diagnostics, Saint Paul, MN) in accordance with published recommendations at 5, 11, and 19 Hz (Oostveen et al., 2003). During resting measures, participants were seated, wearing a nose clip, and were asked to maintain tidal breathing while their cheeks were held firmly by an investigator. Data used for analysis comprised a minimum 10-breath collection period with respiratory rate also reported.

#### 2.4.4 Exhaled nitric oxide measurements

ExNO was measured in accordance with American Thoracic Society/European Respiratory Society recommendations (American Thoracic, SS. European Respiratory, 2005). Participants completed a deep inhalation followed by a slow, steady exhalation against a counter-pressure until one of the handheld ExNO measurement devices (FeNObreath™, MGC Diagnostics, Sorinnes, Belgium and NIOX Vero^®^ handheld ExNO, Circassia, Morrisville, NC) registered a complete measurement (approximately 10 s), with ExNO concentration measured in parts per billion (ppb). ExNO measurements were measured in triplicate at each time point, and the average was used for statistical analysis.

### 2.5 Statistical analysis

Statistical and graphical analyses were conducted using SPSS (v25, Chicago, IL) and Prism 10.0™ (v10.6.0, GraphPad Software, LLC). Given this cohort was an opportunity sample, an *a priori* sample size calculation was not performed. Normality of distribution was assessed prior to determining appropriate statistical tests. Mixed effects analysis with Dunnett’s *post-hoc* pairwise comparison (repeat comparisons against pre-race) was performed to evaluate the effects of time and race among all racers. All statistical tests were two-tailed with significance set to alpha < 0.05 and adjusted *p*-values presented where appropriate. Results are reported as mean ± standard deviation (SD) unless otherwise stated.

## 3 Results

Racer demographics and anthropometrics are presented in **Table 1**. UTMB racers were taller and had greater lean muscle mass compared to HK and CCC racers; all other demographics and anthropometrics were not different between the three racer groups. Twenty-six participants across the three races (HK, *n* = 11; CCC, *n* = 5; UTMB, *n* = 10) returned for testing 24 h post-race.

**Table 1:** Demographic data of Hong Kong 100 (HK), Courmayeur-Champex-Chamonix (CCC), and Ultra-Trail du Mont Blanc (UTMB) participants from pre- to post-races.

Race	All	HK	CCC	UTMB
n	57	18	17	22
Female (%)	8 (14%)	1 (6%)	4 (23.5%)	3 (13.0%)
Age (years)	40.5 ± 10.2	39.6 ± 9.7	41.06 ± 1	40.9 ± 10.6
Height (cm)	175.5 ± 7.2	174.4 ± 6.0	172.8 ± 6.8	178.4 ± 7.5†
Body mass (kg)	69.9 ± 8.3	70.3 ± 7.3	66.9 ± 9.7	71.7 ± 7.7
Lean body mass (kg)	55.7 ± 7.6	51.9 ± 3.0	53.7 ± 12.2	61.5 ± 7.6*†
Body fat (%)	15.0 ± 6.0	15.8 ± 6.0	15.9 ± 6.7	14.5 ± 4.5
Race duration (h)	27.4 ± 11.3	17.1 ± 4.0	21.0 ± 4.0	40.1 ± 4.8*†
Race distance (km)	128.2 ± 35.2	99.0 ± 4.1	101.0 ± 1.0	171.5 ± 0.0*†

Statistical significance was set to alpha <0.05: * significance from HK, ^†^ significance from CCC. Results are presented as mean ± standard deviation.

Statistical significance (*p* < 0.05) is as follows: * denotes significance from HK, ^†^ denotes significance from CCC.

### 3.1 Spirometry and respiratory muscle strength measurements

**Figure 2:**
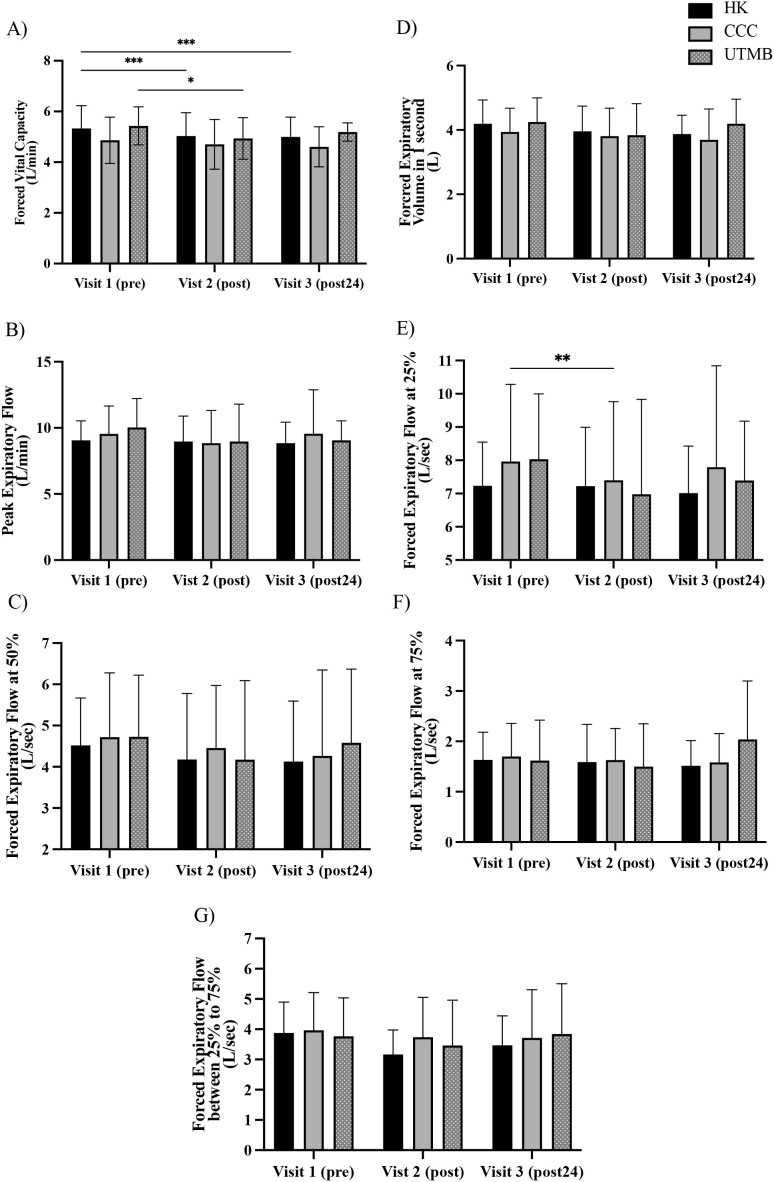
Results from pulmonary function measurements pre-, post-, and 24 h post-ultramarathon race. Results are reported across three races: Hong Kong 100 (HK), Courmayeur-Champex-Chamonix (CCC), and Ultra-Trail du Mont Blanc (UTMB). **(A)** Forced vital capacity (FVC); **(B)** peak expiratory flow (PEF); **(C)** forced expiratory flow at 50% of FVC (FEF_50_); **(D)** forced expiratory volume in 1 s (FEV_1_); **(E)** forced expiratory flow at 25% of FVC (FEF_25_); **(F)** forced expiratory flow at 75% of FVC (FEF_75_); **(G)** forced expiratory flow between 25% and 75% of FVC (FEF_25–75_). Results from mixed effects analysis with Dunnett’s *post-hoc* correction. Statistical significance (*p* < 0.05) is as follows: * denotes significant (*p* < 0.01) difference from pre-race measurements, **p < 0.001, and ***p < 0.0001. Visit 1 (pre-race), Visit 2 (immediately post-race, 1–4 h post), and Visit 3 (18–24 h post-race).

Mixed models evaluating the effects of time and race for spirometry measurements (across all three races) demonstrated significant effects of time for FVC, FEV_1_, PEF, FEF_25–75_, FEF_25_, and FEF_50_ (all *p* < 0.05) (**Figures 2A–G**). While many post-race spirometry measures exhibited tendencies toward reductions, compared to pre-race, statistically significant reductions were only observed for FVC and FEF_25_, of which only the reduction in FVC persisted 24 h post-race (**Figures 2A–G**).

**Figure 3:**
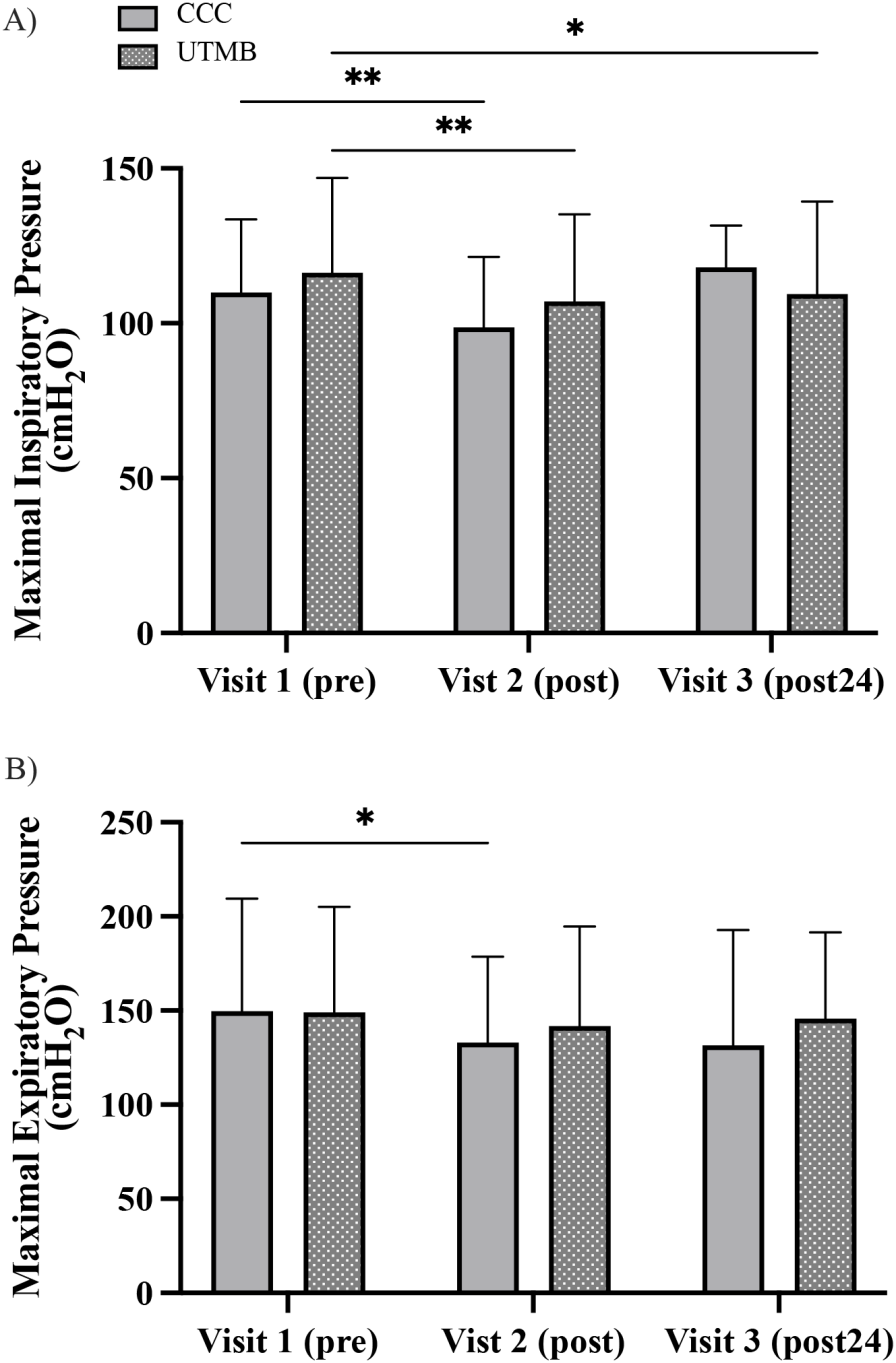
Results from respiratory muscle strength pre-, post-, and 24 h post-ultra-marathon race. Results are reported for two races: Courmayeur-Champex-Chamonix (CCC) and Ultra-Trail du Mont Blanc (UTMB). **(A)** Maximal inspiratory pressure; **(B)** maximal expiratory pressure. Results from mixed effects analysis with Dunnett’s *post-hoc* correction. Statistical significance (*p* < 0.05) is as follows: * denotes significant (*p* < 0.01) difference from pre-race measurements, ** *p* < 0.001, and *** *p* < 0.0001. Visit 1 (pre-race), Visit 2 (immediately post-race, 1–4 h post), and Visit 3 (18–24 h post-race).

For respiratory muscle strength (CCC and UTMB racers only), there was a significant effect of time observed for both MIP and MEP (both *p* < 0.01). MIP demonstrated significant reductions immediately post-race compared to pre-race (for both CCC and UTMB racers, *p* = 0.002 and *p* = 0.001, respectively) with elevations compared to pre-race observed at post-24 h (**Figure 3A**). Furthermore, a significant interaction effect (race × time) was also observed for MIP (*p* = 0.022). MEP demonstrated significant (*p* = 0.013) reductions immediately post-race compared to pre-race only for the UTMB race (**Figure 3B**).

### 3.2 FOT measurements

**Figure 4:**
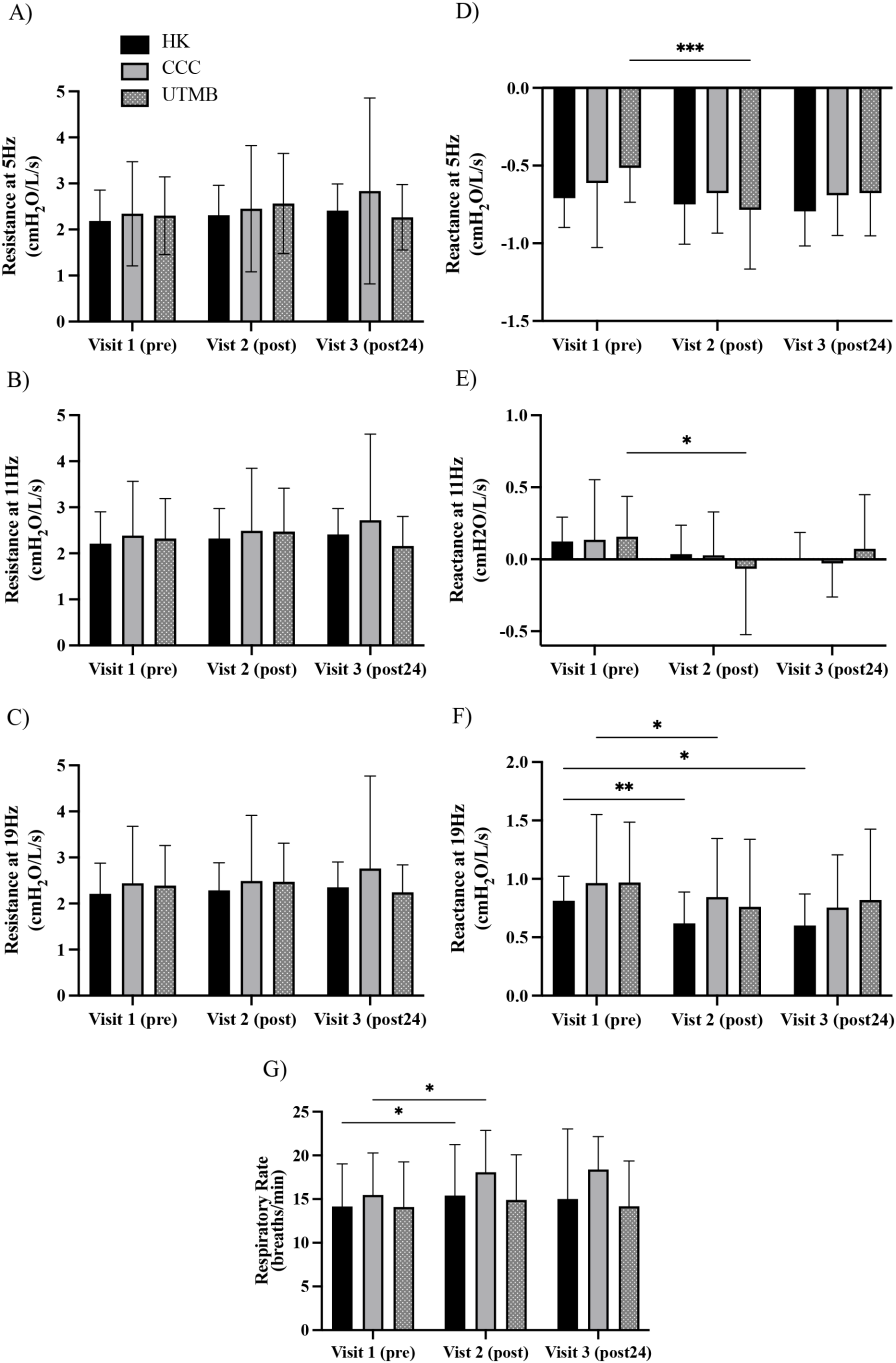
Airway resistance (R) and reactance (X) from forced oscillatory technique (FOT) pre- and post- (immediately and 24-hours) ultramarathon race. Resistance and reactance were reported across 5 **(A, D)**, 11 **(B, E)**, and 19Hz **(C, F)** frequencies. **(G)** Respiratory Rate. Results are reported across three races: Hong Kong 100 (HK), Courmayeur-Champex-Chamonix (CCC), and Ultra-Trail du Mont Blanc (UTMB). Results for mixed effects analysis with Dunnett’s post-hoc correction are marked with statistical significance (p<0.05) denoted by: *denotes significant (*p*<0.01) difference from pre-race measurements. ** *p*<0.001 and *** *p*<0.0001. Visit 1 (pre-race), Visit 2 (immediately post-race, 1–4 hours post), and Visit 3 (18–24 hours post-race).Airway resistance (R) and reactance (X) from forced oscillatory technique (FOT) pre- and post (immediately and 24 h)-ultramarathon race. Resistance and reactance were reported across 5, 11, and 19 Hz frequencies. Results are reported across three races: Hong Kong 100 (HK), Courmayeur-Champex-Chamonix (CCC), and Ultra-Trail du Mont Blanc (UTMB). Results for mixed effects analysis with Dunnett’s *post-hoc* correction are marked with statistical significance (*p* < 0.05): * denotes significant (*p* < 0.01) difference from pre-race measurements, **p < 0.001, and ***p < 0.0001. Visit 1 (pre-race), Visit 2 (immediately post-race, 1–4 h post), and Visit 3 (18–24 h post-race).

Mixed models showed no effects of time or race (or interaction) on airway resistance (R) at any frequency (R_5_, R_11_, or R_19_, all *p* > 0.05) (**Figures 4A–C**). By contrast, a significant effect of time was observed for airway reactance (X) at all frequencies (X_5_, X_11_, and X_19_, all *p* < 0.05; **Figures 4D–F**). A significant effect of time (*p* = 0.002) was also observed for respiratory rate during FOT measures, with significant increases observed immediately post-race for HK and CCC races (*p* = 0.042 and *p* = 0.024, respectively; **Figure 4G**).

### 3.3 Exhaled NO measurements

**Figure 5:**
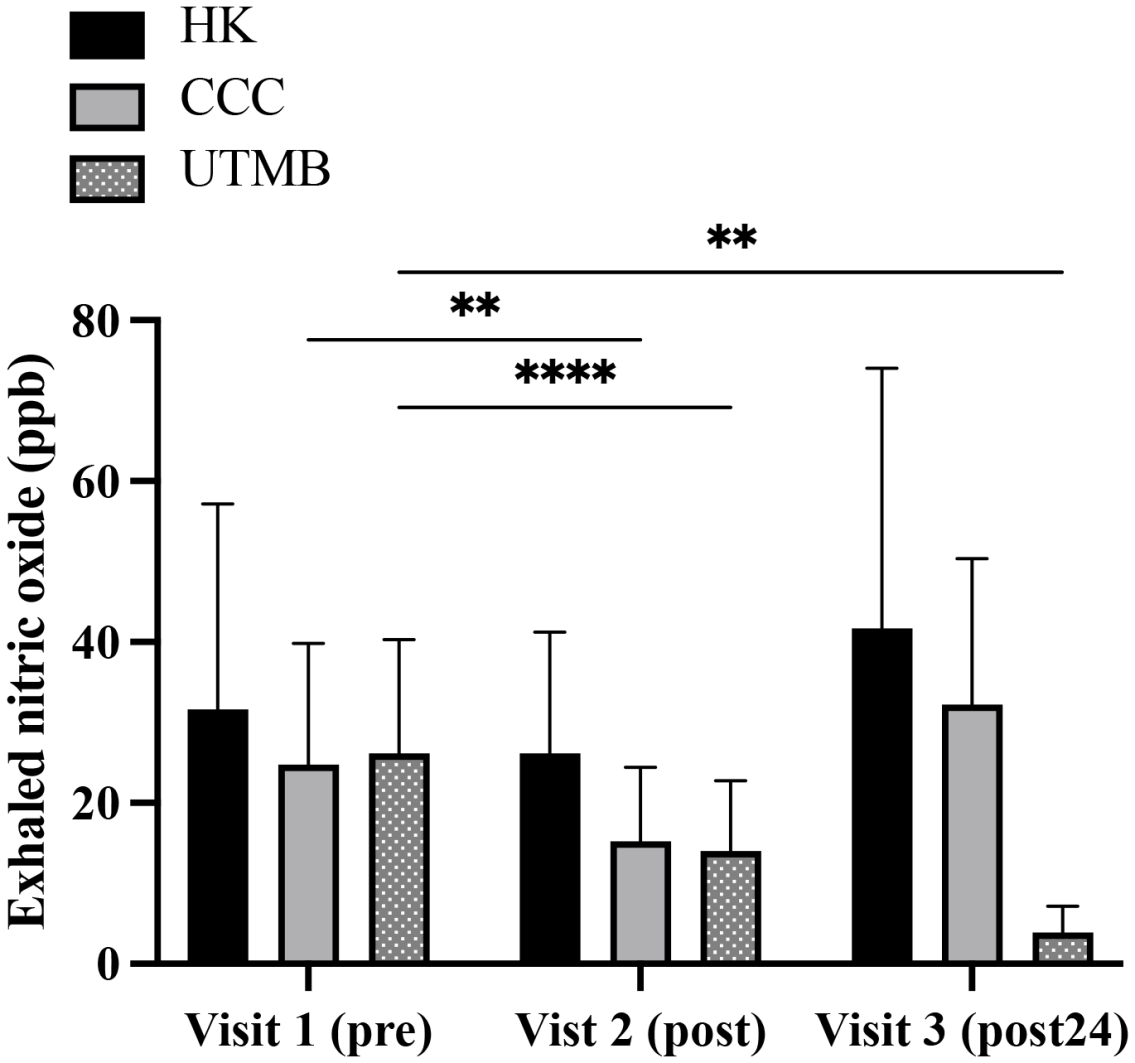
Results from mixed effects analysis for exhaled nitric oxide (ExNO). Results are reported across three races: Hong Kong 100 (HK), Courmayeur-Champex-Chamonix (CCC), and Ultra-Trail du Mont Blanc (UTMB). Results from mixed effects analysis with Dunnett’s *post-hoc* correction. Statistical significance (*p* < 0.05) is as follows: * denotes significant (*p* < 0.01) difference from pre-race measurements, **p < 0.001, and ***p < 0.0001. Visit 1 (pre-race), Visit 2 (immediately post-race, 1–4 h post), and Visit 3 (24 h post-race).

At baseline, nine participants demonstrated ExNO levels that would be classified as severe airway inflammation (>50 ppb). Results from mixed effects analysis for ExNO demonstrated significant effects of time (*p* = 0.004), race (*p* = 0.002), and interaction (time × race, *p* = 0.020) (**Figure 5**).

## 4 Discussion

This study aimed to characterize the changes in lung function in athletes following three different ultramarathon races that varied in elevation gain and distance. We observed significant reductions in airway impedance and lung function metrics post-race among ultra-marathon racers. These reductions persisted up to 24-h post-race and were similar across the difference races despite the variable distance and environmental conditions.

### 4.1 Overall effect of ultra-marathon on airway obstruction, mechanical properties, and lung function post-race

To our knowledge, this is the first study to utilize FOT to measure obstruction and mechanic properties of the lung pre- and post-ultra races. FOT offers the benefit of being a more sensitive way of measuring obstruction and mechanical properties of the lung, as it is not dependent on respiratory muscle strength, fatigue, and participant effort, unlike spirometry. Resistance (R) measured by FOT reflects forward pressure of conducting airways (Oostveen et al., 2003), interpreted at airway obstruction. The current study’s findings observed no evidence of airway obstruction (R at 5, 11, and 19 Hz), but observed a decline in the mechanical or elastic properties (reactance) of the peripheral (X_5_), intermediary (X_11_), and central airways (X_19_), of those who competed in an ultra-marathon race. There are mixed reports on whether ultra-marathon racers may or may not experience air flow obstruction and air trapping and/or peripheral lung impairment/obstruction post-race (Empey, 1972; Mahler and Loke, 1981; Vernillo et al., 2015; Wüthrich et al., 2015). A previous report estimated a prevalence of airflow obstruction and air trapping (defined by a reduction of FEV_1_ over 10%), at 39.0% (Wüthrich et al., 2015) of racers post-race, while others have not reported obstructions (Vernillo et al., 2015). It is speculated that airflow obstruction was not likely a valid explanation for the reductions in lung function, but rather the changes in the elastic properties of the airways, interstitial edema, or respiratory muscle weakness (Harms et al., 1997; Sharma and Brown, 2007). The likely explanation for the expiratory and inspiratory muscle fatigue reported in this study and others (Wüthrich et al., 2015) was a diminished post-race inspiratory capacity.

The observed declines in reactance suggest that ultra-marathon racers experience reductions in lung compliance or how effectively the peripheral airways can be ventilated due to the elastic properties (X_5,_ X_11_, and X_19_) of the airways. One previously presented hypothesis that is supported by our findings for the reduction/limitation, observed by a reduced FEV_1_, in expiratory function is from a loss of lung elasticity and not expiratory fatigue (Hayes and Kraman, 2009; Vernillo et al., 2015). One previous report reported a reduction in both R_5_ and X_5_ after a marathon run (Gibson-Alves et al., 2022); however, this study only observed changes in X_5,_ X_11_, and X_19_ following ultramarathon races. The etiology of these effects of ultra-marathon exposure on peripheral, intermediary, and central airway mechanical properties should be further investigated in terms of narrowing and inflammation markers in the lung.

The current study observed significant pre- to post-race reductions in FVC, FEV_1_, FEF_25–75_, PEF, ExNO, MIP, and MEP among ultra-marathon racers, although not all decrements were to the severity of previous reports (Warren et al., 1989; Ross et al., 2008; Vernillo et al., 2015; Wüthrich et al., 2015). Previous studies investigating the effects of ultra-marathon running on lung function reported conflicting findings. Indeed, some but not all have reported before to after race decreases in FVC and FEV_1_, and PEF, and MVV (Mahler and Loke, 1981; Warren et al., 1989; Rogers et al., 2002; Ross et al., 2008; Vernillo et al., 2015; Wüthrich et al., 2015; Martinez-Navarro et al., 2021). Previous research has observed a significant reduction in respiratory muscle strength following an ultra-marathon, specifically in both MIP (−19%) and MEP (−21%) (Wüthrich et al., 2015) (the current study had 18% and 4% reductions) from pre- to post-race of a previous weather-shortened 101-km UTMB (5,862 m elevation gain) (Wüthrich et al., 2015), as well as an 18% reduction in MIP following a treadmill marathon (Ross et al., 2008). Respiratory muscle fatigue is speculated to occur via a reduced Ca^2+^ release from the sarcoplasmic reticulum and/or overextension of muscle fibers that cause damage to the sarcomeres (Jones, 1996). Although not directly tested in our study, we can suggest a mechanical contribution to this fatigue through changes in airway mechanical properties that caused a reduction in lung function.

Nitric oxide (NO) is a mediator of homeostasis throughout the body (Dweik et al., 2011) and can be synthesized by the airway epithelium and measured in one’s exhaled breath (ExNO), typically as a marker of airway inflammation (Thornadtsson et al., 2017). Within the pulmonary system, NO mediates dilation of the airways and pulmonary vasculature to help balance supply and demand (Kaczmarek et al., 1996; Coggins, 2007). This study evaluated changes in ExNO as it may be affected by the environmental conditions of the race completed. ExNO was significantly decreased from pre-race to immediately post-race (CCC and UTMB). It has been reported that ExNO decreases post-marathon races, potentially in adaptation to strenuous exercise and increased oxygen uptake (Thornadtsson et al., 2017); however, ExNO has been shown to not be a marker of systemic endothelial vascular NO production (Sartori et al., 1999). Reports involving exercising and trekking in the cold (Stensrud et al., 2016) and in high-altitude environments (Brown et al., 2006) resulted in an acute decrease in ExNO. Colder temperatures have been reported to affect post-exercise recovery of ExNO more than ambient temperature and are suggested to reduce ExNO due to vasoconstriction of the airway as NO production is involved with dilation (Brown et al., 2006; Thornadtsson et al., 2017).

It is important to note that the cohort of participants did consist of eight former smokers and three participants with allergies/asthma; however, only one of the former smokers fell into the higher ExNO level (>50 ppb) pre-race and therefore did not affect baseline measures. It has been stated previously that the availability of ExNO is negatively associated to reduced lung function (FEV_1_) (Sierra et al., 2019), but no pre-race lung function values reported any limitations.

### 4.2 Overall effect of ultra-marathon on airway obstruction, mechanical properties, and lung function 24 h post-race

The current study observed a reduction that persisted 24 h post-race of central airway impedance (X_19_), FVC, FEV_1_, FEF_25–75_, and FEF_50_ from pre-race measures. This is contrary to the limited reporting of lung function fully recovering 24 h post-race (Maron et al., 1979; Ross et al., 2008); however, previous reports have pertained to marathons as opposed to ultra-endurance events. The time course of recovery observed in the current study suggests that lung function may have been influenced by airway mechanical airway properties/impedance.

### 4.3 Differing responses based on ultra-marathon race

The UTMB race was a longer race than both HK and CCC races, both CCC and UTMB were at higher elevations than HK, and CCC had a greater elevation change than HK. These differences may explain the race- and time-dependent effects observed in this study (refer to **Figure 5**), which were largely consistent with the previous analysis (Stewart et al., 2024). The minor discrepancies observed here (i.e., increase in ExNO at post-24 h for HK and CCC) following the mutually observed reductions immediately post-race are most likely attributable to the differing number of participants between these analyses (Stewart et al., 2024). Ultra-marathon characteristics warrant further investigation to determine what factors cause larger decrements in lung function and impedance, as there are a multitude of other confounding variables that may be of influence [i.e., race intensity (relative and absolute), ascent/descent profile, time spent at high altitudes, sleep, race conditions, age, sex, temperature, humidity, air quality, etc.].

### 4.3 Limitations

Some limitations of the current study include a dropout rate of 20% of our initial recruitment, which was due to injuries or the inability to meet the time requirements per station; however, a dropout rate of 20% is expected at these types of events. Another limitation of this study was that the post race metrics were not measured immediately and consistently following the race, but rather ranged from within 2 h post race. Additionally, only 46% of racers returned for 24 h post-race.

## 5 Conclusion

Participants who competed in an ultra-marathon experienced a decrease in lung function, ExNO, respiratory muscle fatigue, and mechanical properties of the lung peripherally, intermediately, and centrally from pre- to post-race. Lung function metrics and intermediary and central airway mechanical property decrements were not resolved 24 h post-ultra-marathon. Additionally, the observed reductions were similar among the different ultramarathon races.

## Data Availability

The original contributions presented in the study are included in the article/supplementary material. Further inquiries can be directed to the corresponding author.
